# Juice Powders from Rosehip (*Rosa canina* L.): Physical, Chemical, and Antiglycation Properties

**DOI:** 10.3390/molecules28041674

**Published:** 2023-02-09

**Authors:** Aleksandra Hendrysiak, Jessica Brzezowska, Nancy Nicolet, Dimitri Bocquel, Wilfried Andlauer, Anna Michalska-Ciechanowska

**Affiliations:** 1 Department of Fruit, Vegetable and Plant Nutraceutical Technology, The Faculty of Biotechnology and Food Science, Wrocław University of Environmental and Life Sciences, Chełmońskiego 37, 51-630 Wrocław, Poland; 2 Institute of Life Technologies, School of Engineering, University of Applied Sciences and Arts Western Switzerland (HES-SO Valais Wallis), Rue de l’Industrie 19, 1950 Sion, Switzerland

**Keywords:** *Rosa canina* L., juice, pasteurization, drying, carriers, fruit powder, color, phenolics, carotenoids, antioxidants, glycation

## Abstract

Fruits from rosehip (*Rosa canina* L.) are gaining popularity due to their content and profile of bioactive components. Rosehip is distinct for its antioxidant, immunomodulatory, and anticancer properties. However, the abundance of these bioactives led to a tart taste, resulting in its consumption mainly in processed form. Due to microbiological safety, pasteurization is the preferred way of processing, which affects the chemical properties of the juice. A promising approach to improve acceptability of rosehip’s physical properties, while preserving its bioactive compounds and adding health-promoting benefits, is to enrich the rosehip juice with functional carriers before drying. The influence of the carrier type (maltodextrin, inulin, trehalose, palatinose) and drying technique (spray- and freeze-drying) on the physical, chemical, and antioxidant properties of pasteurized, and non-pasteurized juice powders was examined in this study. In addition, the ability of powders with functional carriers to inhibit protein glycation was evaluated. Spray drying led to products with improved physical properties in relation to freeze-drying. The addition of carrier substances significantly influenced the antioxidant capacity determined by TEAC ABTS and FRAP methods, whereby the application of inulin and palatinose retained antioxidant capacity better than the frequently used maltodextrin. Moreover, rosehip juice powders showed a promising ability to inhibit protein glycation.

## 1. Introduction

Rosehip describes the accessory fruits of the various *Rosa* species which differ in terms of basic chemical components and phytonutrients [1]. *Rosa canina* L. gained special attention due to the presence of numerous biologically active compounds present in the fruit, including phenolics and carotenoids, amongst others [2], resulting in health benefits, such as antioxidant, antibacterial, antidiabetic, anti-inflammatory, immunomodulatory, and anti-obese properties [3,4]. Rosehip is rarely consumed fresh due to its sour taste and hard structure of the fruit, where hairy achenes are enclosed by the red pericarp [5,6,7]. Thus, rosehip fruits are usually processed into juice, puree, or dried products [1,6] that are further used for the production of other foods, including nectars [8] and jams [9]. Usually, such production requires the addition of sugar that affects the nutritional and sensory attributes of rosehip products. This approach further avoids the limitations of fruit seasonality and expiry date of fresh products. One of the other ways to preserve the bioactives present in rosehip is the production of fruit powders [7]. However, to obtain products that may be easily re-solubilized in water, only rosehip juice should be used. The processing of fruits, which includes juicing and pasteurization, alters the chemical composition of the fruit-based matrix composed of sugars, organic acids, pectins and bioactive compounds [10]. The transformation of juice into powder form requires the application of carrier substances with a high glass transition temperature, that enable the procurement of powders from such a complicated matrix. Carriers may also prevent the degradation of the bioactive compounds [11]. Maltodextrin is usually the carrier of choice for powder production, due to its availability and desired technological properties [12]. However, its high glycemic index (185-105) negatively affects the nutritional properties of the powder [13]. Foods with a high glycemic index contribute to the risk of lifestyle diseases due to the accumulation of advanced glycation end products (AGEs) in the body. Numerous studies indicated that food-derived AGEs pose a serious health risk, in the context of type-2 diabetes and atherosclerosis. It has been suggested that it is possible to inhibit the accumulation of dietary AGEs in the body by adjusting the diet accordingly [14]. In this context, carriers are sought to enable the drying process while enriching the powder with functional properties. One of these functional carriers is inulin, having prebiotic properties, a low calorific value (1.5 kcal/g), and a low glycemic index [15,16]. This fructan-type carbohydrate is a natural dietary fiber. Another example of a low glycemic index carrier is the naturally sourced sugar palatinose, which regulates glucose metabolism and improves insulin response [17]. An ingredient with similar physical properties to palatinose is trehalose, a non-reducing sugar, that may decrease the formation of Maillard reaction products during the drying process [18]. Consequently, more research focusing on developing new forms of AGEs inhibitors is needed [19]. This study investigates the antiglycation properties of fruit powders gained with different functional carriers, that can influence their biological properties. However, depending on the type of carrier, different interactions between selected bioactives present in the rosehip juice may occur [12]. Such alterations are also influenced by the selection of drying techniques and parameters applied. Until now, freeze-drying was considered as a technique with the slightest impact on bioactive compounds. More recently, spray drying, resulting in a similar quality of powders, has been recommended [20,21].

Consequently, the aim of the study was to evaluate the influence of the pasteurization, carrier type, and drying techniques on the physical, chemical, and antioxidant properties of rosehip juice powders. In addition, the study aimed at assessing the ability to inhibit the formation of advanced glycation end products (AGEs) by fruit-based powders.

## 2. Results and Discussion

### 2.1. Rosehip Juice Powder Production by Freeze- and Spray Drying

In the current study, powders from rosehip juice were produced with and without the addition of carriers. Powders obtained without carriers were considered controls and were only obtained by freeze-drying. It was unfeasible to obtain powders without carriers from juice by spray drying due to their sugar and organic acid content, leading to a sticky product. The addition of carriers into the liquid feed enabled the production of fruit powders by spray drying. In the study, for the first time palatinose was used as a carrier during the powdering process of rosehip juice; it was due to its functional properties, i.e., a low glycemic index [22]. For all palatinose-added samples subjected to spray drying, the inlet and outlet temperatures were lowered because of the glass transition temperature of palatinose (approx. 62 °C) [22]. For palatinose samples, spray drying with an inlet air temperature above 140 °C was not feasible (data not shown). Thus, an inlet temperature of 130 °C was chosen for drying of all samples (Table 1).

### 2.2. Physical Properties 

#### 2.2.1. Moisture Content (MC)

No significant differences were observed in the MC between powders from pasteurized and non-pasteurized juice, regardless of the carrier addition (Table 2). In line with Michalska and Lech [23], and Caliskan and Dirim [24], freeze-dried powders had an approx. 2-times higher MC when compared to spray-dried products, except samples with inulin. The addition of inulin led to a lower MC when compared to other carriers [25]. A similar observation was noted by Michalska-Ciechanowska et al. [26], who reported lower MC values in freeze-dried cranberry juice powders with inulin. This suggests that inulin may be linked to a different water-holding capacity, which can be modified by an appropriately selected drying technique used for powder production [25].

#### 2.2.2. Water Activity (*a_w_*)

The *a*_w_ of all analyzed powders was below 0.4 (Table 2), which alluded to the biochemical and microbiological stability of these products [27,28]. The pasteurization of the juice before drying had no influence on the water activity of the control samples. However, the drying techniques applied strongly affected the water activity of the powders obtained. Lyophilized powders had approx. 46% higher *a*_w_ values when compared to spray-dried powders. As anticipated, an increase in the drying temperature during spray drying reduced the water activity and the MC. The strong correlation between MC and *a_w_* (*r* = 0.9217) was indicated as noted previously, e.g., for powders from blackcurrant [29] and chokeberry pomace [18]. Presumably, this was linked to a more porous structure of freeze-dried powders [27]. However, the lowest value among the analyzed powders was recorded for the lyophilized sample with inulin. Michalska-Ciechanowska et al. [26] reported a similar observation explaining this phenomenon by the interaction between the carrier and juice during the freezing of the solution, before lyophilization. It is possible that inulin could contribute to the stronger binding of water molecules by the rosehip juice powder [30].

#### 2.2.3. Color and Browning Index (BI)

The differences in the color of powders produced from rosehip juices are presented in Table 2. The L* parameter value ranged from 63.41 ± 5.35 (non-pasteurized, freeze-dried juice with trehalose) to 91.81 ± 0.22 (non-pasteurized juice powder obtained by spray drying with inulin). An addition of the carrier increased the L* coordinate value by an average of approx. 6%, regardless of the drying technique used for powder production. The L* values were, on average, 12% higher for powders obtained by spray drying when compared to freeze-drying. A similar observation was made by Michalska et al. [31]. The exceptions were powders with inulin, which had no significant differences between drying techniques. The presence of inulin (x = 87.10) resulted in the brightest rosehips powders, while palatinose resulted in the darkest products (x = 78.81). The differences in the brightness of the powders depended on the type of carrier used, and could be explained by the interaction between the juice matrix and the carriers [21]. Moreover, lower values of the L* parameter for the lyophilized variants were caused by a higher MC, showing a strong negative correlation (*r* = −0.6915) between these two variables.

Browning often occurs during drying of plant-based products, leading to an increase in the redness of the dried merchandises [32]. The a* parameter values ranged from 0.90 ± 0.16 (spray-dried, a non-pasteurized juice with maltodextrin) to 8.11 ± 0.27 (freeze-dried, pasteurized juice with palatinose). Analyzing only the influence of the drying technique, freeze-drying allowed for obtaining almost 3-times higher values of coordinate a* than spray drying. The biggest difference between the drying techniques was observed for the non-pasteurized juice powder with maltodextrin. Spray drying resulted in 8.3-times greater decrease in red pigments than freeze-drying. When using inulin as a carrier, powders showed a similar level of redness, regardless of the drying technique. However, the addition of inulin resulted in the lowest average a* values, which may point its weakest ability for preservation of red pigments [26].

The highest values of the b* parameter were recorded for freeze-dried powders, independent of the carriers applied. Values of freeze-dried powders were approx. 38% higher than for spray-dried ones. It was concluded that the powders obtained by lyophilization contained more yellow pigments among the analyzed samples. This phenomenon may be explained by the presence of native substances from the raw material, such as carotenoids, which are responsible for the yellow-orange color and likely behave differently at elevated temperatures.

The BI was applied to determine the color changes due to browning [33]. In general, the highest values were recorded for powders obtained by freeze-drying. An exception were powders with the addition of inulin, in which, regardless of the drying technique, the BI values were at the same level. However, the differences between obtained results were similar, meaning that the drying and the addition of the carrier did not significantly affect the color change towards browning. 

### 2.3. Phenolics and HMF

Five phenolic compounds were quantified in rosehip juice powders: (+)-catechin, gallic acid, hydroxybenzoic acid, flavonol quercetin and its glycoside, rutin (Table 3). 

In pasteurized juice powders produced without carrier addition (control), the total content of identified phenolics was 17% higher when compared to non-pasteurized juice. A similar observation was noted by Oliveira et al. [34], who pointed out that pasteurization of peach resulted in an increase in the content of selected phenolics due to their increased extractability after heat treatment. In general, the addition of maltodextrin, trehalose, and palatinose (20%; *w*/*w*) to the juice before drying (regardless of application of pasteurization and drying technique) resulted in a lower content of phenolics by 50% when compared to controls. The highest content of these compounds was noted for powders with inulin. Maltodextrin, trehalose, and palatinose similarly influenced the retention of phenolics in powdered products that were, on average, approx. 40% lower when compared to inulin. Therefore, the addition of inulin to rosehip juice did not degrade the phenolic compounds. This result contrasts the observations reported by Michalska-Ciechanowska et al. [12] for chokeberry products. The addition of inulin to chokeberry extracts led to a degradation of the sum of identified phenolics in powders after freeze drying. This phenomenon confirmed that, depending on the presence of specific types of phenolic compounds, the addition of carriers (regardless of the drying techniques) affected their retention or degradation to a different extent. 

Among all analyzed phenolics, the dominant compound present in rosehip powders was (+)-catechin (85.1% of all compounds), the content of which was the highest in the powders obtained without carrier addition. Pasteurization affected its content, as a higher (+)-catechin level was noted in the case of pasteurized juice powders. It is a known effect that thermal processes increase phenolic content due to a liberation of matrix-bound phenolic constituents [35]. Among carriers added to the juice before drying, inulin influenced the preservation of (+)-catechin to the highest extent. Previously, Michalska-Ciechanowska et al. [12] indicated that inulin used to produce chokeberry powders caused a significantly higher content of (+)-catechin than maltodextrin. Thus, the selection of an appropriate carrier for the drying of fruit-based products is of high importance. The phenolic content was similar between freeze- and spray-dried powders, suggesting that both can be successfully applied for rosehip powders production. 

The second group of phenolic compounds quantified in rosehip juice powders were phenolic acids (12.2% of all phenolics), among which the presence of gallic acid (8.8%) and hydroxybenzoic acid (3.4%) was confirmed. The highest content of these components was indicated for non-pasteurized and pasteurized juice powders gained without carrier addition. Contrary to (+)-catechin, the presence of carriers in pasteurized juice powders did not influence the content of gallic acid. The addition of inulin before the drying of non-pasteurized juice led to higher retention of gallic acid. The content of hydroxybenzoic acid followed the pattern of (+)-catechin. Powders produced without carrier addition had an approx. 45% higher content when compared to the average for the carrier-added counterparts, excluding those with inulin. As with (+)-catechin, inulin caused the highest retention of hydroxybenzoic acid, indicating its protective effect during drying.

The last group of phenolics quantified in rosehip juice powders was flavonols (2.7% of all compounds), which included rutin and quercetin. In the case of control samples (no carrier addition, freeze-drying), a 12% higher content of rutin was noted in powders produced from pasteurized juice when compared to non-pasteurized juice products. Similarly to the observation made for (+)-catechin, a thermal process increases the content of selected phenolics, as confirmed by Margean et al. [36], who observed an increase in rutin after pasteurization of red grape juice. Moreover, Astiti et al. [37] proved that rutin has virucidal properties against the influenza A virus H1N1 and α-glucosidase inhibitory activity, so the application of a pasteurization process may be proposed to increase the rutin level in juice. In the case of powders gained from pasteurized juice with carrier addition, the average content of rutin was 30% higher when compared to powders made from non-pasteurized juice, regardless of the drying techniques applied for powder production. The presence of quercetin has been noticed. However, the quantity in the powders was below the limit of quantification.

Rosehip juice powders were analyzed for their contents of 5-hydroxymethyl-*L*-furfural (HMF). However, its content was under the limit of detection (<0.3 mg/kg of powder) (Table 3). This compound is considered as an indicator of food thermal processing. HMF is formed from hexoses as a result of the heating process of products such as fruit, coffee, milk or honey [38]. HMF is probably carcinogenic, which makes its presence in food products undesirable [12]. Earlier, 5-hydroxymethyl-*L*-furfural was identified in powders from cactus fruit juice [39], plum [40], sea buckthorn [21], Japanese quince [38], and chokeberry [12]. The current study showed that neither drying techniques nor carrier type influence its formation in powders.

### 2.4. Carotenoids 

Previously, carotenoids were identified and quantified in rosehip, and the presence of lycopene was confirmed (2.9–35.2 mg/100 g) [4]. However, the chemical composition varied depending on factors including variety, geographic origin, climate conditions, or part of the fruit. Medveckiene et al. [41] identified different carotenoids in the pulp and seeds of rosehip. In the current study, carotenoid analysis made by HPLC-DAD allowed for the quantification of lycopene (140 μg/kg of powder) only in non-pasteurized and lyophilized rosehip juice, without the addition of a carrier (the control sample) (Table 3). This could be explained by the fact that aqueous juice is not a good matrix to solubilize highly lipophilic carotenoids, including lycopene. Moreover, carotenoids undergo degradation under the influence of light and high temperatures, which probably explains the lack of carotenoids in pasteurized juice powders. Lycopene was present in the control powder from non-pasteurized juice, and it was observed that the carriers did not preserve lycopene during drying. Comunian et al. [42] suggest that the encapsulation of fat-soluble compounds, such as lycopene, is best done by an encapsulation with proteins, while carriers such as maltodextrin are recommended for water-soluble compounds, e.g., phenolics.

### 2.5. Total Phenolic Content (TPC)

TPC values of pasteurized control samples were approx. 7% lower than TPC values of non-pasteurized controls (Table 4). However, differences were not significant. An addition of carriers (regardless of the type) to the juice lowered the TPC content down to approx. 61%. Drying techniques had different effects on TPC that were linked to pasteurization. Seerangurayara et al. [43] observed the same phenomenon in freeze-dried date pulp powders, explaining this by the possibility of diluting the phenolics contained in dates by adding carriers. In the case of non-pasteurized juice powders, TPC values were 25.7% higher for samples obtained by spray drying. In contrast, in the case of juice powders subjected to pasteurization, freeze-drying resulted in higher TPC values (22.4%) when compared to spray drying.

### 2.6. Antioxidant Capacity In Vitro

The antioxidant capacity values of controls obtained by freeze-drying (without an addition of carriers) were 8.4% higher for TEAC ABTS, and 4% higher for FRAP in the case of, non-pasteurized juice, when compared to pasteurized product (Table 4).

The TEAC ABTS and FRAP values for the controls were approx. 2-times higher, for non-pasteurized and pasteurized juice powders, respectively, when compared to products with a 20% carrier addition, despite of the type of carrier [12]. Regardless of the drying technique used, the antioxidant capacity values for powders with maltodextrin addition were lower by 30.2% and 34.4%, respectively, for TEAC ABTS and FRAP, compared to powders made with the carriers, i.e., inulin, trehalose or palatinose. These findings were consistent with those reported by Romero-Gonzalez et al. [44] and Michalska-Ciechanowska et al. [12], who showed that fruit powders containing maltodextrin had lower antioxidant properties compared to other carriers. Drying technique did not significantly influence the ability of the powders to scavenge ABTS^•+^ radicals and reduce the ferric-reducing antioxidant potential. The correlation between TEAC ABTS, as well as FRAP, and a sum of identified phenolics (*r* = 0.6247; *r* = 0.6289), total phenolics content (*r* = 0.8527, *r* = 0.8258), was noted. Michalska et al. [40] also found a strong relation between the content of identified phenolics and the antioxidant capacity of plum juice powders.

### 2.7. Antiglycation Properties

In the current study, three stages of antiglycation activity were evaluated by *in vitro* tests (Table 5). In Bovine Serum Albumin (BSA)-glucose and BSA-fructose model systems, the powder reactivity with reducing sugars during glycation was examined. Pasteurized and non-pasteurized control samples (no carrier addition, freeze-drying) did not statistically significantly differ in their ability to prevent the glycation process. The addition of carriers during powder production slightly increased the antiglycation properties of powders, as was the case of BSA-glucose (< 6.5%). However, in the case of BSA-fructose, no significant differences were noted. There were no significant alterations in the powders gained after freeze- and spray drying. Both drying techniques seem to retain the bioactive compounds that inhibited the glycation [26]. The different response of aminoguanidine among the two employed models comes from the different reactivity of the sugars used. Previously, fructose was reported to be about 10-times more reactive than glucose [45], which may explain the observed discrepancies.

The inhibition of the intermediate stage of protein glycation by the rosehip powders was described based on the results gained from the BSA-methylglyoxal (MGO) model system. In comparison to the BSA-glucose and BSA-fructose models, the inhibition of the powders was significantly weaker. Values ranged from 20 up to 43% (Table 5). Statistically significant differences were observed for control samples indicating a decrease of glycation inhibition in pasteurized samples. Regardless of the juice pre-treatment, carrier addition lowered the glycation inhibitory properties of the powders. No significant differences were observed between the samples with the addition of trehalose and palatinose. However, with maltodextrin, glycation inhibition was lower for freeze-dried, pasteurized juices powders compared to spray-dried products. Powders with the addition of inulin showed statistically significant differences between both drying techniques and the effect of pasteurization. Interestingly, the lowest inhibition among the powders with the addition of carriers was recorded for spray-dried, pasteurized juice with inulin (20.43% ± 0.31), and the highest inhibition for spray-dried powders with inulin from non-pasteurized juice (35.13% ± 1.01). Inulin-type fructan applied for powders preparation might inhibit the formation of AGEs, and therefore further improve their antiglycation properties. Previously, Zhao et al. [46] reported antiglycation activity for fructans and pectins obtained from *Polygonatum* spp. Therefore, it can be speculated that this is one of the possible mechanisms that create antiglycation potential. Nevertheless, this trend was not noted for all variants of powders with inulin addition. This confirms the multiple interactions of the fruit-based matrix and the carriers during the drying of the juices.

The *L*-arginine–MGO model system evaluates specific reactions between methylglyoxal and the guanidine group of arginine forming fluorescent argpyrimidine [47]. The results indicated the weakest inhibition of this glycation stage among all models used. The control samples (freeze-dried juice without the addition of a carrier) did not differ statistically significantly, which indicated that pasteurization had no effect on glycation inhibition. As in the case of the BSA-MGO model, carrier addition to rosehip juices reduced the protein glycation ability of the powders to 57% (Table 5). There were no statistically significant differences in the application of pasteurization, drying techniques or the type of carrier. The weakest anti-glycation ability was observed for powders obtained with an addition of maltodextrin as a carrier.

To sum up, all obtained rosehip juice powders inhibited the protein glycation. However, their activities drastically varied in the four model systems. The differences resulted from different reaction mechanisms depending on the model matrix. Those differences were linked to the composition and content of the bioactives, mainly phenolics present in rosehip juices [48]. Among all glycation stages, the highest correlation was observed for the *L*-arginine–MGO model system with TPC, and the sum of identified phenolics (*r* = 0.8767, *r* = 0.6655). This proved the influence of phenolic compounds on the formation of fluorescent AGEs, as a result of the reaction of MGO with the guanidine group of arginine [47]. A strong correlation between the *L*-arginine–MGO model system and selected phenolic acids, e.g., gallic acid (*r* = 0.8518) and hydroxybenzoic acid (*r* = 0.7777) was confirmed and indicated their ability to inhibit the reaction of methylglyoxal with arginine. Research made by Mesías et al. [49] and Ho et al. [50] confirmed that gallic acid is a good inhibitor of AGE formation. Moreover, the study showed a strong correlation (*r* = 0.6444) between gallic acid and the MGO-BSA system, confirming the ability to inhibit the middle stage of the glycation reaction.

### 2.8. Principal Component Analysis (PCA)

To uncover the hidden patterns in the data set and identify the relationships between observations and variables, principal component analysis (PCA) was used.

**Figure 1:**
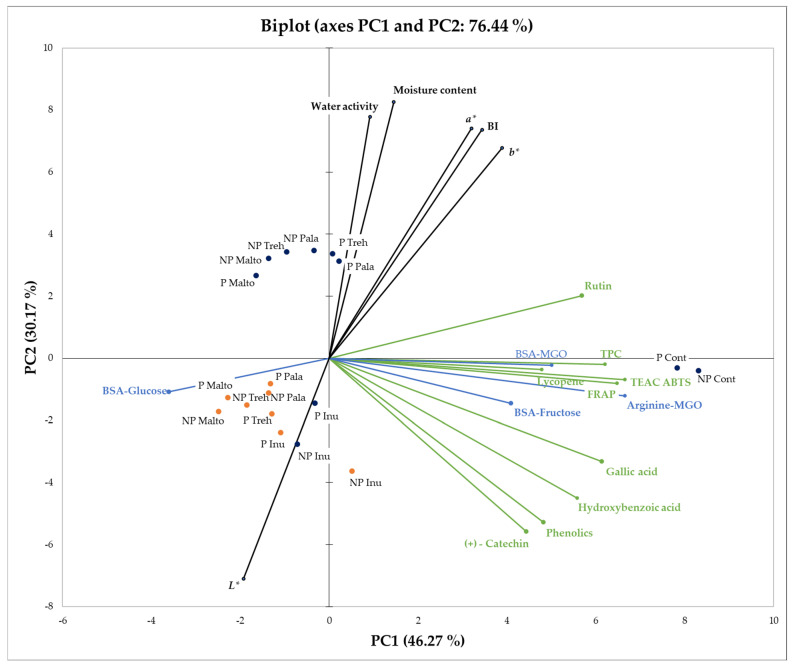
Principal Component Analysis (PCA) biplot of the first two principal components for the rosehip juice powders. PCA indicates how pasteurization of rosehip juice, drying technique (freeze- and spray drying) and application of carrier (no carrier (control), maltodextrin, inulin, trehalose, palatinose) relate to each other; P—pasteurized juice, NP—non-pasteurized juice, Cont—control, Malto—maltodextrin, Inu—inulin, Treh—trehalose, Pala—palatinose, BI—browning index, TPC—Total Phenolics Content, TEAC ABTS—Trolox Equivalent Antioxidant Capacity by ABTS assay, FRAP—Ferric Reducing Antioxidant Potential, FD—freeze-drying marked as navy blue dots, SD—spray drying marked as orange dots.

The score plot of PC1 versus PC2 explained 76.44% (PC1 = 46.27% and PC2 = 30.17%) of all the variation of the experimental dataset (Figure 1). PCA analysis showed that the samples can be clustered into three major groups: (1) controls (non-pasteurized and pasteurized), (2) freeze-dried samples, except inulin (non-pasteurized and pasteurized) and (3) spray-dried products and freeze-dried powders with inulin produced from non-pasteurized and pasteurized juices, causing all powders with this carrier to be in one group. The last group was similar in terms of its ability to inhibit the BSA-glucose model of antiglycation properties and lightness (L*). Both control powders were associated with antiglycation activities evaluated in BSA-fructose, BSA-MGO, and *L*-arginine–MGO models, antioxidant capacity, as well as with all identified phenolics and lycopene. Interestingly, PCA analysis revealed a close relationship between the carotenoid and the powders’ ability to inhibit BSA-MGO-originated advanced glycation products. Finally, freeze-dried powders from the second group were similar in terms of most of their physical attributes, especially water activity and moisture content. This indicates that freeze-drying moderates these parameters considerably more strongly than spray drying.

## 3. Materials and Methods

### 3.1. Material

About 70 kg of rosehip (*Rosa canina* L. fruits) was purchased from local orchard (Szkółka Drzew, Krzewów Owocowych i Róż, Kostrzyn Wielkopolski, Poland) in September 2020. Fruits were sorted, washed, frozen (−18 °C) and stored until juicing.

### 3.2. Chemicals and Reagents

The ABTS (2,2′-azino-bis(3-ethylbenzothiazoline-6-sulfonic acid)) diammonium salt, potassium peroxodisulfate, 2,4,6-tris(2-pyridyl)-s-triazine, Trolox^®^, aminoguanidine hydrochloride, methylglyoxal solution, L-arginine, hexane, 5-hydroxymethyl-*L*-furfural, BHT (2,6-Di-tert-butyl-4-methylphenol), (+)-catechin, rutin, quercetin, D-glucose, citric, L-ascorbic, oxalic, gallic and hydroxybenzoic acid were purchased from Sigma-Aldrich (Switzerland). Acetone and trifluoroacetic acid were bought from Acros Organics (France). Methanol and acetonitrile were obtained from Fine Chemicals (Netherlands), ethyl acetate from Alfa Aesar (Germany). Acetic and sulfuric acids were from Thermo Scientific (Germany). Lycopene, β-carotene, lutein and zeaxanthin were bought from Extrasynthèse (France) and absolute ethanol from Alcosuisse (Switzerland). Fructose and saccharose were obtained from Merck (Germany).

Folin and Ciocaulteu reagent, sodium dihydrogen phosphate mono-hydrate, di-sodium hydrogen phosphate anhydrous and sodium azide were purchased from Chempur (Poland), BSA (Bovine Serum Albumin) from Pol-Aura (Poland). The pectinase Pectinex Ultra SP-L was obtained from Novozymes (Denmark).

Deionized water (Milli-Q purification system, BlancLabo, Switzerland) was used for chromatography.

All reagents used were of analytical grade or higher. All solvents were of HPLC grade.

### 3.3. Juicing

The fruits were cut with rotating knives of a STEPHAN Universal Machine (Type UM SK 60), first, with 50% of maximum speed for 150 s, then at maximum speed for 90 s. The cut fruits reached a temperature of 0 °C at the end of the cutting process. For enzymation, the fruits were split into two batches of 27 kg and transferred into a 100 L double-walled heatable barrel. Water (9 L) and 41 mL of the enzyme preparation (Pectinex Ultra SP-L, Novozymes) were added to each batch and warmed up to 50 °C for 16 h. After this enzymation step, both purees obtained were joined and pressed with a hydraulic Bucher press (Type TPZ 7), applying a pressure gradient up to 180 bar. From the 54 kg of rosehip, 45.6 kg of a cloudy juice (84.4% yield), with 18.28 °Bx and a pH of 3.49, was obtained. The warm, cloudy juice was clarified with a centrifuge (AlfaLaval Type Clara 20, flow 100 L/h, backpressure 1.8 bar). Finally, 43.2 L of juice and 2 L of sludge were obtained.

Half (about 20 L) of the juice was pasteurized with a tubular heat exchanger (APV, Delta 4.50.1), at a flow of 100 L/h and a temperature of 88 °C for 20 s. The juice was directly filled (T > 70 °C) into 5 L bag-in-box containers. The other half of the juice (about 20 L) was stored at −18 °C in the freezer.

The obtained juices (pasteurized, non-pasteurized) were frozen before preparation of solution submitted to drying.

### 3.4. Freeze- and Spray-Drying

Approximately 300 mL of the non-pasteurized juice (17.4 ± 0.2 °Bx) and the pasteurized juice (17.2 °Bx ± 0.1) were mixed with 20% (*w*/*w*) of maltodextrin (Pepees S.A., Łomża, Poland), inulin (Beneo-Orafti, Oreye, Belgium), trehalose (Hayashibara Co., Okayama, Japan) and palatinose (PST-N, isomaltulose; Beneo-Palatinit GmbH, Mannhein, Germany). The mixture of each juice was freeze-dried (FreeZone, Labconco Corp., MO, USA) for 24 h under reduced pressure of 65 Pa (temperature of the chamber and heating plate: −60 °C/ +24 °C) and spray-dried (Mini Spray dryer B-290 Advanced, Büchi, Flawil, Switzerland; two-fluid nozzle atomizer with inside diameter of 1.5 mm; spray drying parameters were adjusted to inlet air temperature of 130 °C as presented in Table 1). Addition of palatinose to the rosehip juices required lower aspirator values due to the stickiness issue in the drying chamber. A freeze-dried juice without the addition of carrier served as a control sample.

### 3.5. Physical Properties

#### 3.5.1. Moisture Content (MC) and Dry Matter (DM)

The MC was performed in duplicate (*n* = 2) by the vacuum-oven method, according to Figiel [51] at 80 °C for 24 h and was expressed as %. The DM was calculated by the difference between MC and 100%.

#### 3.5.2. Water Activity (*aw*)

The *aw* value was measured using the water activity meter Dew Point (Water Activity Meter, 4TE, AQUA LAB, Pullman, WA, USA) at 25 °C in duplicate (*n* = 2).

#### 3.5.3. Color and Browning Index (BI)

The color of the powders was analyzed in triplicate (*n* = 3) (Minolta Chroma Meter CR-400 colorimeter; Minolta Co. Ltd., Osaka, Japan), according to the CIE L*a*b* system. The BI was calculated according to the equation proposed by Maskan [52].

### 3.6. Antioxidant Capacity In Vitro

The extraction was performed according to the procedure described by Michalska-Ciechanowska et al. [18]. The antioxidant capacity of powders was examined by TEAC ABTS [53] and FRAP [54] assays, using a Synergy H1 spectrophotometer (BioTek Instruments Inc., Santa Clara, CA, USA). The determination was made in duplicate (*n* = 2) and the results were expressed as mmol Trolox Equivalent (TE) per 100 g of dry matter (DM).

### 3.7. Total Phenolic Content (TPC)

The TPC of the rosehip juice powders was examined in 80% methanol extracts using the Folin–Ciocalteu method, as described by Gao et al. [55] and modified by Horszwald and Andlauer [56]. A Synergy H1 spectrophotometer (BioTek Instruments Inc., Santa Clara, CA, USA) was used. Gallic acid was used as a standard and results (*n* = 2) were expressed as g gallic acid equivalents (GAE)/100 g DM.

### 3.8. Sample Preparation for Antiglycation Assays

Extracts were prepared according to the procedure proposed by Wang et al. [47]. To prepare the extracts, each variant of juice powders (*n* = 2) was weighed in duplicate (*n* = 2) using an analytical balance (AS 110/C/S, Radwag, Poland). The prepared samples were dissolved in 10 mL of a 30% methyl alcohol solution with 1% formic acid and sonicated for 15 min. The extracts were stored at 4 °C for 24 h and then sonicated again (15 min). Centrifuged extracts were transferred to laboratory heart flasks and evaporated to dryness using a rotary vacuum evaporator type Unipan 350P (Warsaw, Poland). The samples were dissolved in a sodium phosphate buffer (PBS, 50 mmol/L; pH 7.4; with 0.02% sodium azide), in the same volume as the extraction medium. Dissolved samples were directly used for the determination of anti-glycation properties.

### 3.9. Antiglycation Assay

The analysis of the antiglycation properties of powders was carried out, with slight modifications, according to the procedure described by Wang et al. [47].

#### 3.9.1. BSA-Glucose/Fructose Model

This model determines the ability of the obtained plant powders to inhibit all stages of protein glycation. A glucose/fructose solution (1.5 mol/L, 0.5 mL) was added to 0.5 mL of extracts dissolved in a phosphate buffer solution (PBS) and incubated at 37 °C for 2 h at 110 rpm. After this time, the BSA solution (30 mg/mL, 0.5 mL) was added to each sample and incubated again under the same conditions for 7 d. A PBS sample without the addition of plant extracts was used as a blank. An aminoguanidine solution (30 mmol/L) was used as a positive control instead of the extracts dissolved in PBS. The blank and positive control were subjected to the same procedure as the samples with the plant extracts. Fluorescence was measured using a Synergy H1 spectrophotometer (BioTek Instruments Inc., Santa Clara, CA, USA), at an excitation wavelength of λ = 340 nm and an emission wavelength of λ = 420 nm. The analysis was performed in quadruplicate (*n* = 4) and the results were expressed as a percentage of the AGE inhibition according to the equation of Wang et al. [47]:

$$\mathrm{Percentage}\;\mathrm{inhibition}=\left(1-\frac{\mathrm{Fluorescent}\;\mathrm{intensity}\;\mathrm{with}\;\mathrm{inhibitor}}{\mathrm{Fluorescent}\;\mathrm{intensity}\;\mathrm{without}\;\mathrm{inhibitor}}\right)\times \;100\;\%$$

#### 3.9.2. BSA-MGO Model

This model determines the ability of the obtained plant powders to inhibit the intermediate stages of protein glycation. A methylglyoxal solution (MGO) (60 mmol/L, 0.5 mL) was added to 0.5 mL of extracts dissolved in PBS and incubated at 37 °C for 2 h at 110 rpm. Further steps were analogous to the BSA-glucose/fructose model. Fluorescence was measured using a Synergy H1 spectrophotometer (BioTek Instruments Inc., Santa Clara, CA, USA), at an excitation wavelength of λ = 340 nm and an emission wavelength of λ = 380 nm. The analysis was performed in quadruplicate (*n* = 4) and the results were expressed as a percentage of the AGE inhibition according to the equation in the BSA-glucose/fructose model.

#### 3.9.3. *L*-arginine–MGO Model

This model determines the ability of the obtained plant powders to inhibit a specific reaction when methylglyoxal reacts with the guanidino group of arginine. The MGO solution (60 mmol/L; 0.5 mL) was added to 0.5 mL extracts dissolved in PBS and incubated at 37 °C for 2 h at 110 rpm. After this time, arginine solution (60 mmol/L; 0.5 mL) was added to each sample and incubated again under the same conditions for 7 d. The PBS (without the addition of *Rosa canina* powder extracts) was used as a blank. An aminoguanidine solution (30 mmol/L) served as a positive control instead of the extracts dissolved in PBS. The blank and positive control were subjected to the same procedure as the samples with the plant extracts. Fluorescence was measured with a Synergy H1 spectrophotometer (BioTek Instruments Inc., Santa Clara, CA, USA), at excitation wavelength λ = 340 nm and emission wavelength λ = 380 nm. The analysis was performed in quadruplicate (*n* = 4) and the results were expressed as a percentage of the AGE inhibition according to the equation in the BSA-glucose/fructose model.

### 3.10. Carotenoid Chromatographic Analysis

During the whole extraction process, the samples were protected from light and the temperature did not exceed room temperature. Portions of 1 g of fruit powder were weighed and extracted in the ultrasonic bath at 0 °C (ice bath) for 10 min with 1 mL hexane:acetone (50:50; *v*:*v*) solvent mixture containing 0.1% BHT. The mixture was centrifuged at 4 °C for 10 min (4400 rpm). The extraction was carried out once. The supernatant was filtered through filtered tips into an Eppendorf tube and concentrated under nitrogen flow to obtain a final volume of 40 µL. The solution was transferred into HPLC vials and stored at 4 °C until analysis.

Chromatographic analysis was performed on an Agilent 1220 Infinity LC Series (Santa Clara, CA, USA). The apparatus was equipped with an autosampler, a quaternary pump and a column oven (25 °C). It was coupled to a diode array detector (DAD). A 250 × 4.6 mm, 5 µm Luna C18 100 Å column, protected by a Phenomenex guard column (2 × 4.6 mm), was employed. The mobile phase consisted of two eluants: acetone (A), and methanol (B). The injection volume was 5 µL. The gradient started with 5% of A and A was increased to 55% within 15 min. This setting was held for 1 min, before decreasing A to 5%. The flow rate was set at 1 mL/ min. The column was re-equilibrated between injections for 4 min with the initial conditions. Lycopene showed a retention time of 7.6 min and β-carotene of 10.4 min. Quantification was done at λ = 450 nm by an external calibration curve in the concentration range of 0.10–5.0 mg/L for lycopene and 0.15–10.0 mg/L for β-carotene, respectively. The limit of quantification (LOQ) was determined as a signal to noise ratio of 10. The LOQ was 100 µg/L for lycopene and 150 µg/L for β-carotene, which corresponds to a LOQ of 100 µg/kg for lycopene and 150 µg/kg for β-carotene in the fruit powders. The limit of detection (LOD) was determined as a signal to noise ratio of 3. The LOD was 30 µg/L for lycopene and 50 µg/L for β-carotene, which corresponds to a LOD of 30 µg/kg for lycopene and 50 µg/kg for β-carotene in the fruit powders.

### 3.11. Phenolics and 5-Hydroxymethyl-L-furfural (HMF) Chromatographic Analysis

Portions of 12 mg of fruit powder were weighed and extracted in the ultrasonic bath at room temperature for 10 min, with 120 µL of eluent A. Chromatographic analyses were performed on an Agilent 1220 Infinity LC Series (Santa Clara, CA, USA). The apparatus was equipped with an autosampler, a quaternary pump and a column heater (35 °C). It was coupled to a diode array detector (DAD). A 100 × 2.1 mm, 2.6 µm Kinetex EVO C18 100 Å column, protected by a 2 × 2.1 mm, 2 µm Phenomenex guard column, was employed. The injection volume was 1 µL. The mobile phase consisted of two eluents: water with 0.05% trifluoroacetic acid (A), and acetonitrile with 0.05% trifluoroacetic acid (B), with a flow rate of 0.4 mL/min. The first 16 min, A was at 100%. Then, A was decreased to 95% within 4 min, and after that to 35% within 4 min. This setting was held for 2 min, and in the next 4 min, A was increased to 100%. Before next injection, the column was re-equilibrated for 8 min with 100% of eluent A. 

Phenolic compounds have been identified through their retention time and the UV spectrum compared with commercial standard compounds. Quantification was done by external calibration. Gallic acid (retention time 1.4 min), hydroxybenzoic acid (4.3 min) and catechin (12.1 min) have been quantified at λ = 210 nm, rutin (24.2 min) and quercetin (24.9 min) at λ = 340 nm. Quercetin had an LOQ of 0.1 mg/L, which corresponds to 8 mg/kg of the fruit powders. LODs were determined as a signal to noise ratio of 3 and LOQs with a signal to noise ratio of 10.

HMF was identified through retention time (1.8 min) and its UV spectrum compared with the standard. Quantification was done at λ = 280 nm with external calibration. HMF showed an LOD of < 0.03 mg/L. Consequently, for 12 mg of powders solubilized in 120 µL of eluent A, the LOD was < 0.3 g/kg powder.

### 3.12. Statistical Analysis and Chemometrics

The obtained results were statistically analyzed using the STATISTICA 13 program (StatSoft, Tulsa, OK, USA). Average values were analyzed by the analysis of variance (ANOVA) and the Tukey post hoc test was used (*p* ≤ 0.05). A Pearson’s correlation coefficient was calculated to test the relationship between the selected variables.

Principal component analysis (PCA) was carried out in order to reduce the dimensionality (volume) of the dataset used, while retaining as much data as possible necessary for interpretation (testing). The idea of PCA has been extensively described by Jolliffe and Cadima [57]. PCA was performed using XLSTAT Statistical and data analysis solution [58].

## 4. Conclusions

This study showed the possibility of obtaining rosehip juice powders using lyophilization and spray drying, with the addition of maltodextrin, as well as the less commonly used functional carriers such as inulin, trehalose, and palatinose. Powders obtained from pasteurized and non-pasteurized juice showed no significant differences for moisture content and water activity, independent of the drying technique used and the type of carrier applied. Higher average values of the moisture content and water activity were reported in lyophilized powders compared to spray-dried ones. Higher and significant differences were observed for powders with inulin as a carrier. The color of the powders in most cases depended on the drying technique used. The values of coordinates a*, b* and browning index were the highest in products gained after lyophilization, while for the L* parameter, spray drying resulted in lighter products. In dried rosehip juice, five phenolic compounds were identified: (+)-catechin, gallic acid, hydroxybenzoic acid, the flavonol quercetin and its glycoside, rutin. The addition of a carrier to the juice reduced the sum of identified phenolics in the obtained powders. The carrier addition lowered the phenolic content in the powders compared to powders without a carrier addition. It was observed that neither pasteurization nor the drying technique influenced the total content of phenolics. Among the carriers used, inulin allowed for the highest preservation of individual phenolics, except for gallic acid (in powders obtained from pasteurized juice) and rutin. (+)-Catechin was the dominant phenolic compound in the powders. No HMF was identified in the analyzed products. The carotenoid lycopene was identified only in the lyophilized powder obtained from non-pasteurized rosehip juice, without carrier addition. Powders with the addition of a carrier (regardless of the type) were characterized by significantly lower TPC values, compared to the control samples without any carrier. Addition of carriers resulted in lower antioxidant capacity values, especially when maltodextrin was used, highlighting the influence of the carrier on the chemical properties of powders. All obtained rosehip juice powders were able to inhibit the formation of AGE products. The highest inhibition of the formation of fluorescent AGEs by rosehip juice powders was noted in the BSA-fructose model. Taking the above into consideration, it can be concluded that rosehip juice powder components, including carriers used for their production, are possibly involved in the antiglycative activity of these products.

## Figures and Tables

**Table 1:** Spray drying parameters set up for the preparation of rosehip juice powders in dependence of the carrier used.

Type of Juice	Carrier	Inlet Air Temperature	Outlet Air Temperature	Volume Flow	Feed Flow
Non-pasteurized	Maltodextrin	130 °C	82 °C	26 m^3^/h	4 mL/m^3^
	Inulin		82 °C	35 m^3^/h	
	Trehalose		74 °C	26 m^3^/h	
	Palatinose		77 °C	26 m^3^/h	
Pasteurized	Maltodextrin	130 °C	82 °C	35 m^3^/h	4 mL/m^3^
	Inulin		84 °C	35 m^3^/h	
	Trehalose		83 °C	35 m^3^/h	
	Palatinose		77 °C	26 m^3^/h	

**Table 2:** Moisture content (MC), water activity (*a*_w_), color (CIE L*a*b*) and browning index (BI) of rosehip juice powders obtained after freeze- and spray drying (*n* = 2; average ± standard deviation). (-)—no carrier addition, M–maltodextrin, I–inulin, T–trehalose, P–palatinose, FD—freeze-drying, SD—spray drying, AU—arbitrary units; ^a,b,c,d,e,f,g^—the same letters within a column indicated no statistically significant differences (HSD Tukey test; *p* ≤ 0.05).

Type of Juice	Drying Technique	Carrier	MC	*a* *_w_*	Color			BI
			(%)	(-)	L*	a*	b*	(AU)
Non-Pasteurized	FD	(-)	4.82 ± 0.46 ^d^	0.24 ± 0.01 ^e^	79.57 ± 0.18 ^cde^	6.39 ± 0.04 ^efg^	31.52 ± 0.02 ^g^	0.40 ± 0.00 ^ef^
		M	7.10 ± 0.34 ^f^	0.38 ± 0.00 ^h^	81.50 ± 0.59 ^def^	7.41 ± 0.45 ^gh^	30.30 ± 1.16 ^g^	0.40 ± 0.01 ^de^
		I	2.44 ± 0.35 ^abc^	0.11 ± 0.01 ^b^	89.26 ± 0.13 ^hi^	1.86 ± 0.02 ^a–d^	19.43 ± 0.43 ^cd^	0.35 ± 0.00 ^abc^
		T	6.96 ± 0.01 ^ef^	0.34 ± 0.00 ^g^	63.41 ± 5.35 ^a^	5.32 ± 0.90 ^e^	26.10 ± 2.74 ^f^	0.41 ± 0.01 ^ef^
		P	6.51 ± 0.24 ^ef^	0.30 ± 0.00 ^f^	70.84 ± 0.64 ^b^	7.11 ± 0.42 ^fgh^	30.75 ± 1.32 ^g^	0.42 ± 0.01 ^f^
	SD	M	2.33 ± 0.18 ^abc^	0.18 ± 0.00 ^cd^	90.61 ± 1.11 ^hi^	0.90 ± 0.16 ^a^	13.76 ± 0.96 ^a^	0.34 ± 0.00 ^a^
		I	2.12 ± 0.37 ^ab^	0.11 ± 0.01 ^b^	91.81 ± 0.22 ^i^	1.63 ± 0.02 ^abc^	16.15 ± 0.09 ^a–d^	0.34 ± 0.00 ^ab^
		T	2.71 ± 0.08 ^abc^	0.16 ± 0.00 ^c^	90.75 ± 2.40 ^hi^	2.25 ± 0.86 ^a-d^	16.11 ± 2.04 ^abc^	0.35 ± 0.01 ^ab^
		P	2.37 ± 0.08 ^abc^	0.18 ± 0.00 ^cd^	81.81 ± 0.96 ^def^	1.81 ± 0.02 ^a-d^	15.39 ± 0.11 ^ab^	0.35 ± 0.00 ^ab^
Pasteurized	FD	(-)	4.80 ± 0.35 ^d^	0.24 ± 0.00 ^e^	75.94 ± 1.61 ^c^	7.46 ± 0.27 ^gh^	30.88 ± 0.27 ^g^	0.41 ± 0.00 ^ef^
		M	6.41 ± 0.19 ^ef^	0.37 ± 0.00 ^h^	79.12 ± 1.10 ^cd^	5.75 ± 0.63 ^ef^	25.29 ± 1.99 ^f^	0.38 ± 0.01 ^d^
		I	2.94 ± 0.19 ^bc^	0.07 ± 0.01 ^a^	84.02 ± 0.07 ^efg^	2.83 ± 0.06 ^cd^	23.22 ± 0.18 ^ef^	0.37 ± 0.00 ^c^
		T	6.73 ± 0.06 ^ef^	0.30 ± 0.00 ^f^	75.86 ± 0.26 ^c^	7.89 ± 0.01 ^h^	30.73 ± 0.08 ^g^	0.41 ± 0.00 ^ef^
		P	6.12 ± 0.16 ^e^	0.24 ± 0.00 ^e^	74.82 ± 0.41 ^bc^	8.11 ± 0.27 ^h^	30.98 ± 0.72 ^g^	0.41 ± 0.00 ^ef^
	SD	M	1.81 ± 0.06 ^a^	0.12 ± 0.01 ^b^	86.93 ± 1.48 ^fgh^	3.04 ± 0.36 ^d^	18.40 ± 1.24 ^bcd^	0.35 ± 0.00 ^abc^
		I	2.29 ± 0.13 ^abc^	0.10 ± 0.00 ^b^	83.31 ± 4.84 ^d–g^	1.25 ± 0.07 ^ab^	16.49 ± 0.51 ^a-d^	0.35 ± 0.00 ^ab^
		T	1.79 ± 0.18 ^a^	0.11 ± 0.00 ^b^	89.06 ± 1.29 ^ghi^	2.24 ± 0.34 ^bcd^	17.44 ± 0.67 ^bcd^	0.35 ± 0.00 ^ab^
		P	3.18 ± 0.09 ^c^	0.18 ± 0.00 ^d^	87.77 ± 1.00 ^ghi^	3.04 ± 0.14 ^d^	19.76 ± 0.41 ^de^	0.36 ± 0.00 ^bc^

**Table 3:** The content of identified phenolics (mg/g DM), hydroxymethyl-*L*-furfural (HMF) (mg/g DM) and carotenoids (µg/kg DM) in rosehip juice powders obtained after freeze- and spray drying (*n* = 2; average ± standard deviation). (-)—no carrier addition, M–maltodextrin, I–inulin, T–trehalose, P–palatinose, FD—freeze-drying, SD—spray drying, DM—dry matter, nd—non detected; ^a,b,c,d,e,f,g^—the same letters within a column indicated no statistically significant differences (HSD Tukey test; *p* ≤ 0.05).

Type of Juice	Drying Technique	Carrier	Catechin	Gallic Acid	Hydroxybenzoic Acid	Rutin	Sum of Phenolics	HMF	Carotenoids
			(mg/g DM)						(µg/kg DM)
Non-Pasteurized	FD	(-)	2.28 ± 0.19 ^bc^	0.32 ± 0.03 ^d^	0.14 ± 0.01 ^e^	0.07 ± 0.01 ^fg^	2.81 ± 0.24 ^bc^	nd	140
		M	1.10 ± 0.04 ^a^	0.13 ± 0.01 ^a^	0.05 ± 0.00 ^a^	0.04 ± 0.00 ^ab^	1.31 ± 0.05	nd	nd
		I	2.26 ± 0.09 ^bc^	0.21 ± 0.01 ^c^	0.09 ± 0.00 ^c^	0.04 ± 0.01 ^ab^	2.60 ± 0.07 ^bc^	nd	nd
		T	0.95 ± 0.02 ^a^	0.13 ± 0.01 ^a^	0.04 ± 0.00 ^a^	0.03 ± 0.00 ^ab^	1.15 ± 0.03 ^a^	nd	nd
		P	0.98 ± 0.07 ^a^	0.14 ± 0.01 ^a^	0.04 ± 0.00 ^a^	0.05 ± 0.01 ^bc^	1.20 ± 0.08 ^a^	nd	nd
	SD	M	1.33 ± 0.00 ^a^	0.14 ± 0.02 ^ab^	0.05 ± 0.00 ^ab^	0.03 ± 0.00 ^ab^	1.56 ± 0.01 ^a^	nd	nd
		I	2.51 ± 0.06 ^cd^	0.21 ± 0.00 ^bc^	0.09 ± 0.00 ^c^	0.04 ± 0.00 ^bc^	2.84 ± 0.06 ^cd^	nd	nd
		T	1.32 ± 0.00 ^a^	0.13 ± 0.02 ^a^	0.05 ± 0.00 ^ab^	0.02 ± 0.00 ^a^	1.53 ± 0.01 ^a^	nd	nd
		P	1.22 ± 0.04 ^a^	0.14 ± 0.01 ^a^	0.05 ± 0.00 ^a^	0.04 ± 0.00 ^ab^	1.44 ± 0.04 ^a^	nd	nd
Pasteurized	FD	(-)	2.76 ± 0.22 ^d^	0.33 ± 0.05 ^d^	0.11 ± 0.01 ^d^	0.09 ± 0.01 ^ab^	3.29 ± 0.29 ^d^	nd	nd
		M	1.09 ± 0.04 ^a^	0.10 ± 0.01 ^a^	0.04 ± 0.00 ^a^	0.03 ± 0.00 ^g^	1.26 ± 0.05 ^a^	nd	nd
		I	2.10 ± 0.14 ^b^	0.15 ± 0.01 ^abc^	0.07 ± 0.00 ^bc^	0.06 ± 0.00 ^cde^	2.37 ± 0.15 ^b^	nd	nd
		T	1.06 ± 0.08 ^a^	0.10 ± 0.01 ^a^	0.04 ± 0.00 ^a^	0.06 ± 0.00 ^def^	1.26 ± 0.09 ^a^	nd	nd
		P	1.05 ± 0.07 ^a^	0.09 ± 0.01 ^a^	0.04 ± 0.00 ^a^	0.07 ± 0.01 ^efg^	1.26 ± 0.08 ^a^	nd	nd
	SD	M	1.27 ± 0.05 ^a^	0.13 ± 0.00 ^a^	0.05 ± 0.00 ^ab^	0.03 ± 0.00 ^ab^	1.49 ± 0.05 ^a^	nd	nd
		I	2.11 ± 0.11 ^bc^	0.15 ± 0.02 ^abc^	0.09 ± 0.01 ^c^	0.05 ± 0.01 ^bcd^	2.39 ± 0.13 ^bc^	nd	nd
		T	1.29 ± 0.12 ^a^	0.14 ± 0.00 ^ab^	0.05 ± 0.00 ^ab^	0.02 ± 0.00 ^a^	1.51 ± 0.11 ^a^	nd	nd
		P	1.25 ± 0.10 ^a^	0.05 ± 0.00 ^a^	1.25 ± 0.10 ^a^	0.02 ± 0.00 ^a^	1.44 ± 0.10 ^a^	nd	nd

**Table 4:** The total phenolic content (TPC), antioxidant capacity measured by TEAC ABTS and FRAP methods in rosehip juice powders obtained after freeze- and spray drying (*n* = 2; average ± standard deviation). (-)—no carrier addition, FD—freeze-drying, SD—spray drying, DM—dry matter; ^a,b,c,d,e,f,g^—the same letters within a column indicated no statistically significant differences (HSD Tukey test; *p* ≤ 0.05).

Type of Juice	Drying Technique	Carrier	TPC	TEAC ABTS	FRAP
			(g Gallic Acid/ 100 g DM)	(mmol Trolox/ 100 g DM)	
Non-Pasteurized	FD	(-)	6.98 ± 0.71 ^h^	48.58 ± 1.50 ^h^	35.90 ± 0.57 ^d^
		Maltodextrin	2.13 ± 0.14 ^a–e^	15.48 ± 0.13 ^ab^	12.14 ± 1.39 ^ab^
		Inulin	1.84 ± 0.54 ^ab^	22.88 ± 0.90 ^def^	19.00 ± 0.35 ^c^
		Trehalose	1.34 ± 0.21 ^a^	24.45 ± 0.64 ^ef^	21.49 ± 0.35 ^c^
		Palatinose	2.37 ± 0.30 ^a–f^	24.38 ± 0.94 ^ef^	19.50 ± 1.05 ^c^
	SD	Maltodextrin	2.03 ± 0.54 ^abc^	18.29 ± 0.59 ^c^	14.51 ± 0.81 ^b^
		Inulin	2.21 ± 0.32 ^a-e^	22.93 ± 0.49 ^def^	20.18 ± 0.25 ^c^
		Trehalose	2.35 ± 0.21 ^a-g^	24.75 ± 0.02 ^ef^	19.97 ± 0.01 ^c^
		Palatinose	3.74 ± 0.34 ^fg^	24.28 ± 0.14 ^ef^	20.61 ± 0.24 ^c^
Pasteurized	FD	(-)	6.52 ± 0.26 ^h^	44.50 ± 0.35 ^g^	34.46 ± 1.10 ^d^
		Maltodextrin	3.36 ± 0.20 ^efg^	17.10 ± 1.16 ^bc^	13.85 ± 0.51 ^ab^
		Inulin	3.77 ± 0.12 ^g^	21.72 ± 0.05 ^d^	19.30 ± 0.17 ^c^
		Trehalose	3.17 ± 0.07 ^c–g^	22.70 ± 0.16 ^def^	19.21 ± 0.16 ^c^
		Palatinose	3.53 ± 0.27 ^efg^	23.40 ± 0.51 ^def^	20.67 ± 1.19 ^c^
	SD	Maltodextrin	1.90 ± 0.28 ^a–d^	14.30 ± 0.69 ^a^	11.60 ± 0.05 ^a^
		Inulin	2.89 ± 0.16 ^b–g^	22.11 ± 0.23 ^de^	18.83 ± 0.16 ^c^
		Trehalose	2.70 ± 0.48 ^b–g^	23.78 ± 0.34 ^def^	20.31 ± 0.06 ^c^
		Palatinose	3.24 ± 0.26 ^d–g^	22.88 ± 0.24 ^def^	19.24 ± 1.17 ^c^

**Table 5:** Inhibitory effects of rosehip powders on AGE formation in BSA-glucose, BSA-fructose, BSA-methylglyoxal (BSA-MGO) and *L*-arginine–methylglyoxal model at 37 °C for 7 days. Aminoguanidine (10 mM) served as a positive control (*n* = 2; average ± standard deviation). (-)—no carrier addition, FD—freeze-drying, SD—spray drying; ^a,b,c,d,e,f,g^—the same letters within a column indicated no statistically significant differences (HSD Tukey test; *p* ≤ 0.05).

Type of Juice	Drying Technique	Carrier	BSA-glucose	BSA-fructose	BSA-MGO	*L*-arginine–MGO
			Fluorescent AGE Inhibition [%]			
Non-Pasteurized	FD	(-)	60.24 ± 0.99 ^b^	88.00 ± 0.03 ^bcd^	38.03 ± 0.85 ^g^	44.61 ± 0.22 ^c^
		M	62.54 ± 0.65 ^bc^	87.10 ± 0.05 ^be^	30.02 ± 0.60 ^cde^	14.61 ± 0.55 ^ab^
		I	65.54 ± 0.94 ^c^	87.47 ± 0.08 ^bcd^	29.87 ± 1.22 ^cde^	15.64 ± 1.32 ^ab^
		T	63.73 ± 0.50 ^bc^	87.36 ± 0.17 ^bcd^	28.06 ± 0.99 ^c^	18.87 ± 1.54 ^b^
		P	64.40 ± 0.79 ^c^	87.37 ± 0.23 ^bcd^	30.51 ± 0.59 ^c–f^	20.50 ± 0.81 ^b^
	SD	M	62.39 ± 0.12 ^bc^	86.77 ± 0.59 ^b^	31.20 ± 0.29 ^c–f^	18.22 ± 1.29 ^ab^
		I	64.99 ± 0.76 ^c^	88.43 ± 0.66 ^cd^	35.13 ± 1.01 ^fg^	24.01 ± 1.11 ^b^
		T	63.42 ± 1.23 ^bc^	87.52 ± 0.16 ^bcd^	27.85 ± 1.59 ^c^	19.36 ± 0.31 ^b^
		P	64.78 ± 0.33 ^c^	87.60 ± 0.17 ^bcd^	28.57 ± 0.08 ^cd^	22.39 ± 0.17 ^b^
Pasteurized	FD	(-)	62.42 ± 1.07 ^bc^	88.72 ± 0.23 ^d^	43.33 ± 1.06 ^h^	46.75 ± 0.44 ^c^
		M	62.33 ± 0.89 ^bc^	87.07 ± 0.09 ^be^	28.77 ± 0.38 ^cd^	14.78 ± 1.03 ^ab^
		I	64.65 ± 1.42 ^c^	86.87 ± 0.84 ^b^	29.82 ± 4.05 ^cde^	18.04 ± 0.44 ^ab^
		T	64.80 ± 0.15 ^c^	87.64 ± 0.05 ^bcd^	33.38 ± 1.12 ^d–g^	21.74 ± 2.36 ^b^
		P	65.43 ± 0.14 ^c^	87.90 ± 0.00 ^bcd^	31.14 ± 0.08 ^c–f^	20.74 ± 0.65 ^b^
	SD	M	64.51 ± 0.11 ^c^	87.83 ± 0.06 ^bcd^	33.8 ± 1.49 ^efg^	17.42 ± 2.65 ^ab^
		I	64.37 ± 0.89 ^c^	86.97 ± 0.88 ^be^	20.43 ± 0.31 ^b^	23.62 ± 0.97 ^ab^
		T	63.43 ± 0.55 ^bc^	87.82 ± 0.26 ^bcd^	32.61 ± 0.06 ^c–f^	21.40 ± 1.78 ^b^
		P	65.81 ± 0.35 ^c^	88.14 ± 0.09 ^bcd^	33.43 ± 0.62 ^d–g^	22.34 ± 2.13 ^b^

## Data Availability

The data presented in this study are available on request from the three corresponding authors.

## Sample Availability

Samples of the compounds are not available from the authors.
